# Concretizing plan specifications as realizables within the OBO foundry

**DOI:** 10.1186/s13326-024-00315-0

**Published:** 2024-08-20

**Authors:** William D. Duncan, Matthew Diller, Damion Dooley, William R. Hogan, John Beverley

**Affiliations:** 1 University of Florida, Gainesville, FL USA; 2 Simon Fraser University, Burnaby, BC, CA Canada; 3 Medical College of Wisconsin, Milwaukee, WI USA; 4 University at Buffalo, Buffalo, NY USA

**Keywords:** Ontology, OBO foundry, Information artifact ontology, Ontology for biomedical investigations, Common core ontologies, Failed plans, Plan specification

## Abstract

**Background:**

Within the Open Biological and Biomedical Ontology (OBO) Foundry, many ontologies represent the execution of a plan specification as a process in which a realizable entity that concretizes the plan specification, a “realizable concretization” (RC), is realized. This representation, which we call the “RC-account”, provides a straightforward way to relate a plan specification to the entity that bears the realizable concretization and the process that realizes the realizable concretization. However, the adequacy of the RC-account has not been evaluated in the scientific literature. In this manuscript, we provide this evaluation and, thereby, give ontology developers sound reasons to use or not use the RC-account pattern.

**Results:**

Analysis of the RC-account reveals that it is not adequate for representing failed plans. If the realizable concretization is flawed in some way, it is unclear what (if any) relation holds between the realizable entity and the plan specification. If the execution (i.e., realization) of the realizable concretization fails to carry out the actions given in the plan specification, it is unclear under the RC-account how to directly relate the failed execution to the entity carrying out the instructions given in the plan specification. These issues are exacerbated in the presence of changing plans.

**Conclusions:**

We propose two solutions for representing failed plans. The first uses the Common Core Ontologies ‘prescribed by’ relation to connect a plan specification to the entity or process that utilizes the plan specification as a guide. The second, more complex, solution incorporates the process of creating a plan (in the sense of an intention to execute a plan specification) into the representation of executing plan specifications. We hypothesize that the first solution (i.e., use of ‘prescribed by’) is adequate for most situations. However, more research is needed to test this hypothesis as well as explore the other solutions presented in this manuscript.

## Background

Specifications and protocols are critical for coordinating and regulating activities. For example, a *Study Design* may specify the order and timing for adding a reagent, or a medical protocol may provide detailed instructions about how to carry out a cancer treatment regimen. Information about how to perform an activity (e.g., a set of instructions) is distinct from the performance of that activity. The same set of instructions can be executed by many distinct processes with differing results. For example, the information in the same medical protocol may be used to direct the treatments of two different patients, with one patient being cured and the other not.

Relating instructional information to the set of actions during which the instructions were performed, however, is not straightforward. Intuitively, specifications and protocols involve instructions intended to direct a course of action. Ontologically, relating instructions, the abilities of agents to pursue those instructions, and the actual pursuit of those instructions, is rather complex. As an example, developers of the Information Artifact Ontology (IAO)^1^ characterize these relationships and relevant entities as a *Process* in which a *Realizable Entity* that **concretizes**^2^ the *Plan Specification*, a “realizable concretization” (RC), is realized. In other words, for a given protocol, steps in a relevant course of action realize the abilities of some agent, where these abilities ground the protocol. For brevity, we refer to the IAO approach as the “RC-account”, and although the RC-account is formally correct, we hold that it leads to difficult issues about how to represent and (1) when an agent’s^3^ plan^4^ (in the sense of intention) fails to be successfully carried out and (2) when an agent’s plan changes. As a remedy to these issues, we propose the adoption of the Common Core Ontologies^5^ (CCO) [1] **prescribed by** relation, and the addition of classes to represent processes that create an agent’s plan to carry out the information describing what is to be performed.

### IAO Summary

IAO is a domain-neutral ontology for representing the “information content” of information artifacts, such as documents, databases, and digital images [2]. It provides a semantic framework for distinguishing information and activities by extending the Basic Formal Ontology (BFO) [3]. Table 1.

**Table 1:** Depicts the relevant part of BFO’s hierarchy

● *Continuant* ○ *Independent Continuant* ○ *Generically Dependent Continuant* ○ *Specifically Dependent Continuant* ■ *Quality* ■ *Realizable Entity* ● *Disposition*	● *Occurrent* ○ *Process*

At the highest level, BFO distinguishes entities according to whether they are a *Continuant* or an *Occurrent*. An *Occurrent* extends over time and has temporal parts. A *Process* is an *Occurrent* that depends for its existence on some *Material Entity*. For example, a particular occurrence of eating is an instance of *Process* that depends on multiple instances of *Material Entity*, such as what is being eaten and the thing doing the eating.

A *Continuant* lacks temporal parts, endures through time, and participates in instances of *Occurrent*. Instances of *Continuant* are further distinguished according to whether they depend on other entities for their existence. An *Independent Continuant* (*IC*) does not depend on anything for its existence. For example, a particular baseball is an instance of *IC*. It has a particular shape that depends on the baseball for its existence, but the baseball does not depend on the shape for its existence.

Instances of *Specifically Dependent Continuant* (*SDC*) always depend on the same instance or instances of *IC* for their existence. For example, the shape of a baseball is an instance of *SDC* that only exists as long as the baseball exists. The shape is not and cannot be dependent on a different baseball for its existence. More specifically, the shape of the baseball is an instance of *Quality*. A *Quality* is fully manifested whenever it exists. In contrast, an instance of *Realizable Entity* need not fully manifest at any time it exists. For example, many composite dental restoration materials have the *Disposition* to harden when exposed to ultraviolet light but are malleable prior to being used to restore a tooth. The *Disposition* (to harden) of the composite may or may not manifest. For example, if the material is never exposed to ultraviolet light, it will not harden.

Instances of *Generically Dependent Continuant* (*GDC*), a sibling of *SDC*, also depend on an instance or instances of *IC* for their existence. However, unlike an *SDC*, an instance of *GDC* is not required to depend on the same *IC* (or *IC*s) during the course of its existence. Rather, a *GDC* may migrate (or be copied) from one *IC* to another *IC* at different times. For example, a computer virus, an instance of *GDC*, can be copied to multiple computers, and the computer virus continues to exist even if some of the computers delete the computer virus from its system. See Appendix 2 for a more technical description of the differences between specific and generic dependence.

In IAO, information is represented as an instance of *Information Content Entity* (*ICE*), which is a subtype of *GDC*:

*Information Content Entity* = df A *GDC* that is about some thing.^6^

A given *ICE* instance may be shared by multiple entities, with each entity bearing a particular *SDC*, such as a pattern of ink or pixels, that concretizes the *ICE*.

**concretizes**: A relationship between a *SDC* and a *GDC* in which the *GDC* depends on some *IC* in virtue of the fact that the *SDC* also depends on that same *IC*.

**Fig. 1:**
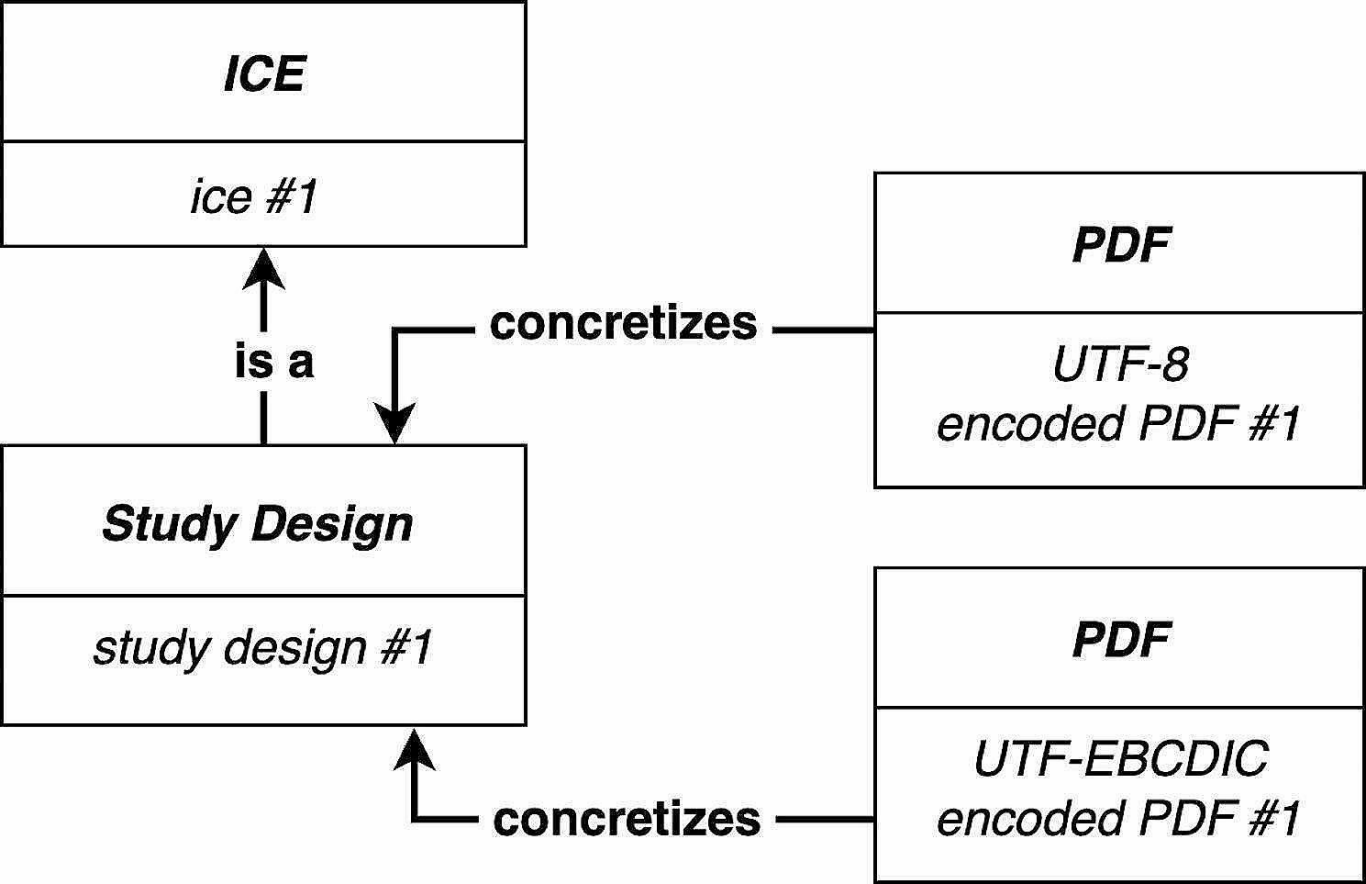
Two differently encoded PDFs concretize the same *Study Design*

For example, a *Study Design*, an instance of *ICE*, may be concretized as a PDF^7^ encoded using UTF-8^8^ on one computer, while on another computer the *Study Design* may be concretized as a PDF encoded using UTF-EBCDIC^9^ (see Fig. 1). The PDFs on each computer concretize the same *ICE* instance, but the *ICE* is concretized, via the PDFs’ encodings, differently in each.

An *ICE* that is about how to perform some actions is defined in IAO as a *Directive Information Entity* (DIE):

*Directive Information Entity* = df An *ICE* whose concretizations indicate to their bearer how to realize them in a process.

A *DIE* that is about specific actions to perform as well as one or more specified objectives is defined as a *Plan Specification*:

*Plan Specification* = df A *DIE* with action specifications and objective specifications as parts that, when concretized, is realized in a process in which the bearer tries to achieve the objectives by taking the actions specified.

For example, the aforementioned *Study Design* is an instance of a *Plan Specification*, and this instance may be concretized in distinct dependent entities across multiple bearers, all bearing the same instance of the *Study Design*. Note that although the definition seems to indicate that the plan specification itself is realized, that is not the case. It is only ever the case that a concretization of the plan specification is realized (in BFO, a *GDC* cannot be realized).

Importantly, IAO’s model representing information about how to perform some set of actions to attain specified objective has been widely adopted within the Open Biological and Biomedical (OBO) Foundry [4], with over 40 ontologies within the OBO Foundry including the term *Plan Specification* (see Appendix 3).

### Motivation for the RC-account

Although neither the formal definitions of *DIE* nor *Plan Specification* formally invoke the RC-account, it is clear that their definitions employ the notion of a realizable concretization, and this notion has been formalized in a number of OBO ontologies. For example, the Ontology for Biomedical Investigations (OBI) [5] defines *Planned Process* as.

A *Process* that **realizes** a plan which is the concretization of a *Plan Specification*.

and is logically equivalent to:^10^

**realizes** some (**concretizes** some *Plan Specification*).

OBI’s *Planned Process* has been imported into at least 60 OBO ontologies (see Appendix 4), and, therefore, these ontologies also indirectly incorporate the RC-account.

**Fig. 2:**
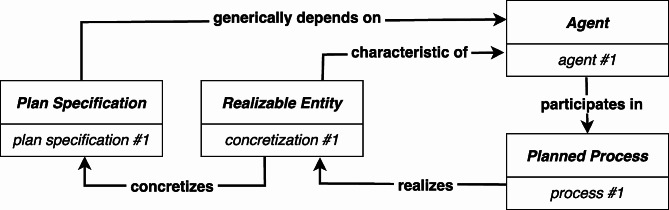
RC-account: *concretization #1*** concretizes ***plan specification #1*, *concretization #1*** characteristic of ***agent #1*, and *process #1*** realizes ***concretization #1*

The motivation for the RC-account is straightforward. It provides a design pattern for relating a *Plan Specification* to the agent that carries out the prescribed actions to the particular *Process* during which the agent performed the actions. An instance of *Realizable Entity* (*concretization #1*) **concretizes** an instance of *Plan Specification* (*plan specification #1*); *concretization #1* is a **characteristic of**^11^ a particular agent (*agent #1*), and an instance of *Process* (*process #1*) **realizes***concretization #1*. The actions specified in *plan specification #1* are executed during *agent #1*’s participation in *process #1* (see Fig. 2):^12^^,^^13^

Thus, data represented using the RC-account design pattern enables ontology developers and users to precisely determine (or query) who executed a particular plan, which actions should have been performed, and the purpose of executing the plan.

## Methods

Although many ontology developers may find the RC-account plausible, we find it lacking in two important ways. First, under the RC-account, it is difficult to provide a robust account of failed plans. Second, it has difficulty representing cases in which an agent’s plan to execute some set of instructions changes. Each issue taken alone casts doubt on the plausibility of the RC-account, and when both issues are considered together, we hold that the plausibility of the RC-account is greatly diminished. Let us look at each issue in turn.

### Failed plans

Providing a coherent account of how a particular *Plan Specification* fails to be executed is an important and necessary condition for any robust ontological theory of how plans are carried out. A plan may fail for several reasons. Extenuating circumstances may prevent the execution from succeeding or the agent responsible for carrying out the plan may have misread (or misremembered the *Plan Specification*). For example, when a surgery fails due to the patient adversely reacting to anesthesia, it is necessary to determine if this was a result of the surgical team failing to ask or remember which medications the patient is allergic to, the anesthesiologist administering the wrong dosage, or an unknown medical condition that caused the patient to react adversely.

**Fig. 3:**
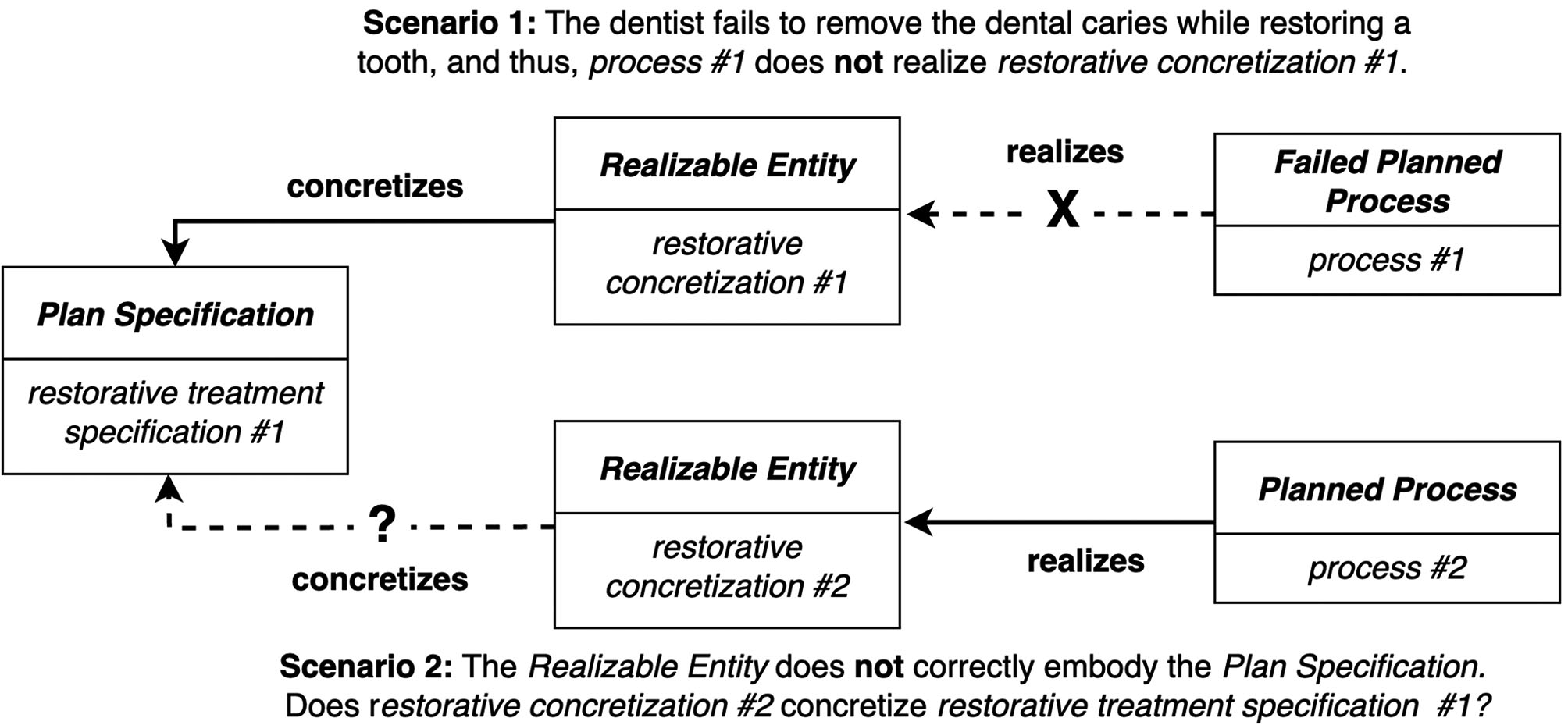
Two scenarios that depict the ways in which a plan can fail under the RC-account. In scenario 1, there is an issue with the *Planned Process*. In scenario 2, there is an issue with the *Realizable Entity*. In both scenarios, the veracity of the RC-account is called into question

Within the framework of the RC-account, the question of whether a failed process can realize a concretized *Plan Specification* poses the following dilemma. On the one hand, if a failed process does not realize a concretized *Plan Specification*, there is not a direct set of relations linking the *Plan Specification* to the agent that executes it during a particular *Process*. And yet, the direct linking between *Plan Specification*, *Agent*, and *Process* is one of the main motivations for the RC-account. For example, when a dentist restores a decaying tooth, removing the existing caries from the lesion is a necessary step during the dental restoration procedure. If the dentist fails to do so, the tooth will continue to decay, and thus, the restoration procedure (an instance of *Planned Process*) will fail to bring about the actions and goals specified by the *Plan Specification* for restoring a tooth (see scenario 1 in Fig. 3). Furthermore, if we allow for a failed process to realize a concretized *Plan Specification* (such as in the example of a failed tooth restoration procedure), we need to provide an account for how a *Realizable Entity* can be realized in a *Process* that does not fully manifest the *Realizable Entity*. In contrast, consider the solubility of a portion of salt as a typical example of *Realizable Entity*. The solubility of the salt is **realized in** the dissolving of the salt in water. However, the whole portion of salt does not have to be dissolved for this realization to occur. If the salt is taken out of the water before the portion is completely dissolved, the solubility is still realized. The execution of a plan is quite different. It involves steps that fulfill goals, and the satisfaction of all the goals completes the plan. In this sense, a dentist’s plan to restore a tooth requires complete realization. The RC-account captures this sense but cannot capture when the dentist’s plan goes awry.

On the other hand, if a plan fails because the concretization is flawed, it is unclear what the nature of the **concretizes** relation should be. If we permit **concretizes** to relate a *Plan Specification* to a *Realizable Entity* that does not correctly represent the actions and goals described by the *Plan Specification*, this entails that extreme misunderstandings or contravening intentions would also count as concretizations. For example, if a dentist’s recollection of how to perform a procedure skips several crucial steps, the dentist’s plan (i.e., realizable concretization) to carry out the misremembered plan would concretize the (correct) *Plan Specification* (see scenario 2 in Fig. 3). Or, if the dentist actively rejects a particular *Plan Specification*, the dentist’s plan not to execute a *Plan Specification* would also be a realizable concretization.

While we recognize that in many situations that an agent’s plan to execute a *Plan Specification* will not be a perfect reflection of the *Plan Specification’s* contents, removing all normative requirements from the **concretizes** relation is not satisfactory.

### Plans change

The relation between a particular *Plan Specification* and an agent’s plan to execute it is not necessarily static. You can read the concretization of a *Plan Specification*, understand the information about how to perform the actions and attain the objectives described within it, but not intend to act on this information. The information content of the *Plan Specification* can remain in your memory for an extended period of time until a particular situation arises that motivates you to form the plan to carry out the *Plan Specification.* However, before acting on your plan, you may decide that this is not the correct course of action as a result of learning new information or a change in circumstance. You, again, no longer intend to execute the *Plan Specification*, and, as before, you retain the information content of the *Plan Specification* in your memory.

**Fig. 4:**
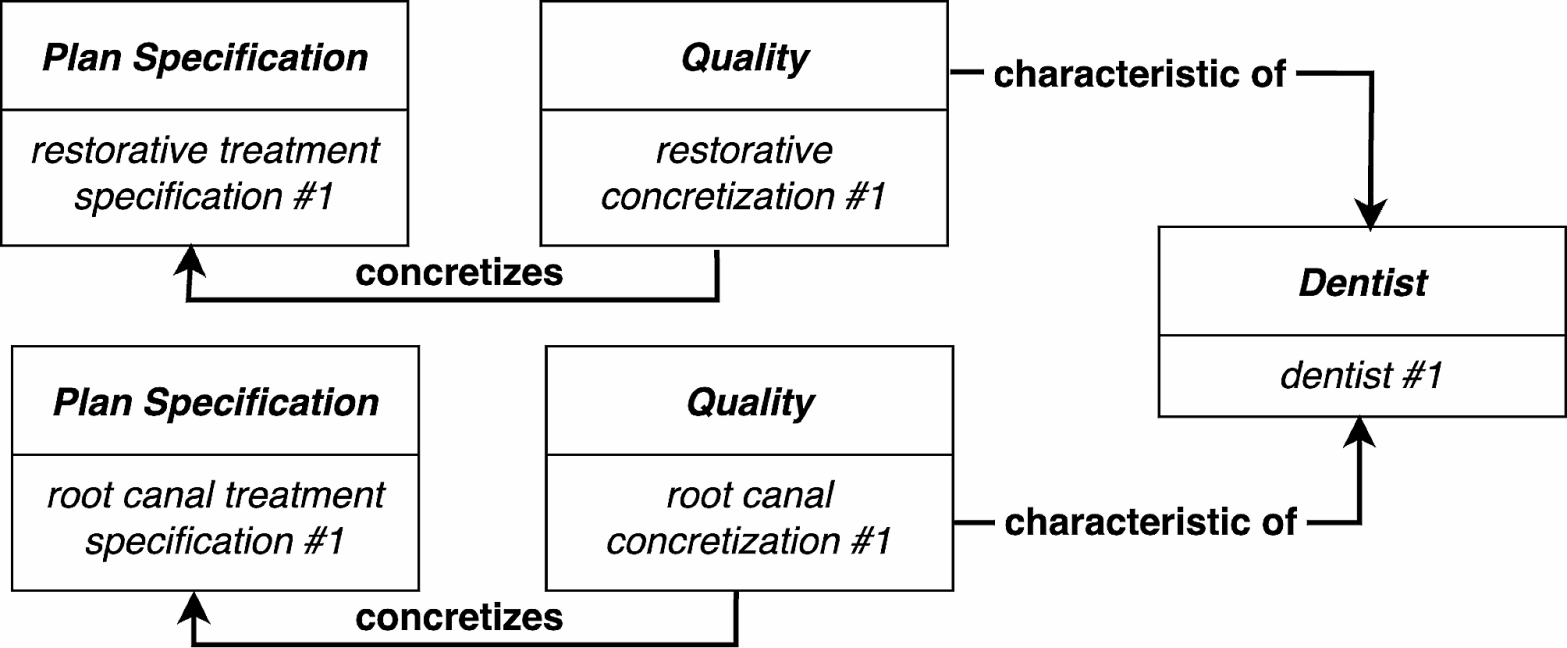
When considering which dental procedure to perform, the dentist bears the concretizations of two particular kinds of *Plan Specification*: information about how to perform a restoration, and information about how to perform a root canal

As a concrete example, let’s consider two kinds of *Plan Specification* that are regularly executed by practicing dentists. Through years of training, a particular dentist retains (in memory) concretizations of the *Plan Specification* for how to perform tooth restorations (a.k.a. fillings) and root canals. Since the dentist does not necessarily plan to carry out these procedures, we represent the concretizations as instances of *Quality* (see Fig. 4^14^^,^^15^):

The plan to perform these procedures comes about as a result of encountering patients who are in need of these treatments. For example, suppose the dentist examines a patient and finds that a large portion of a tooth is severely decayed. The standard of care in such cases permits the dentist to treat the tooth by either (but not both) restoring the tooth using a dental restoration material or performing a root canal. Initially, the dentist decides that the tooth can be saved by performing a restoration. However, after more closely examining a radiograph of the tooth, the dentist decides it is not possible to restore the tooth, and performing a root canal is the better option.

**Fig. 5:**
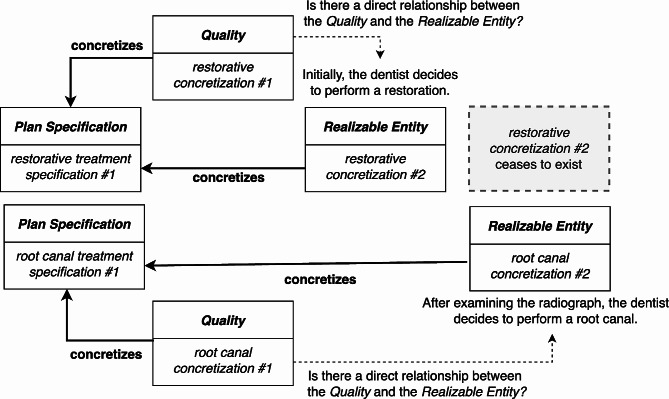
After reassessing the patient, the dentist decides that a root canal is the better treatment. Are there any direct relationships between the *Qualities* and the *Realizable Entities*?

Although it is possible to represent this scenario under the RC-account (see Fig. 5), it leaves open the nature of the relationship between the *Quality* that concretizes the *Plan Specification* and the correlated *Realizable Entity* that also concretizes the *Plan Specification.* Intuitively, one’s plan to carry out a *Plan Specification* requires they make use of information they have stored somewhere in their mind. Under the RC-account, the best ontological representation of this phenomena is via an indirect relation between the particular concretizations.

The issue of changing plans does not necessarily reveal an internal inconsistency with the RC-account. As an agent’s plans change, new realizable concretizations may be created that concretize a particular *Plan Specification*. However, the lack of a more meaningful account of how the concretized *Quality* is related to the realizable concretization is unsatisfying.

## Results

So far, we have discussed failed plans and changed plans as two issues with the RC-account. However, the motivations underlying the RC-account are still pertinent for representing the execution of a *Plan Specification*. We offer the following solutions, compatible with IAO, that will address the issues with failures and changes in plans.

### Prescribed by relation

Our first proposal is that IAO make use of the **prescribed by** relation in the Common Core Ontologies (CCO), which is defined as:

**prescribed by**: x **prescribed by** y iff y is an instance of *Information Content Entity* and x is an instance of *Entity*, such that y serves as a rule or guide for x if x is an *Occurrent*, or y serves as a model for x if x is a *Continuant*.

**Fig. 6:**
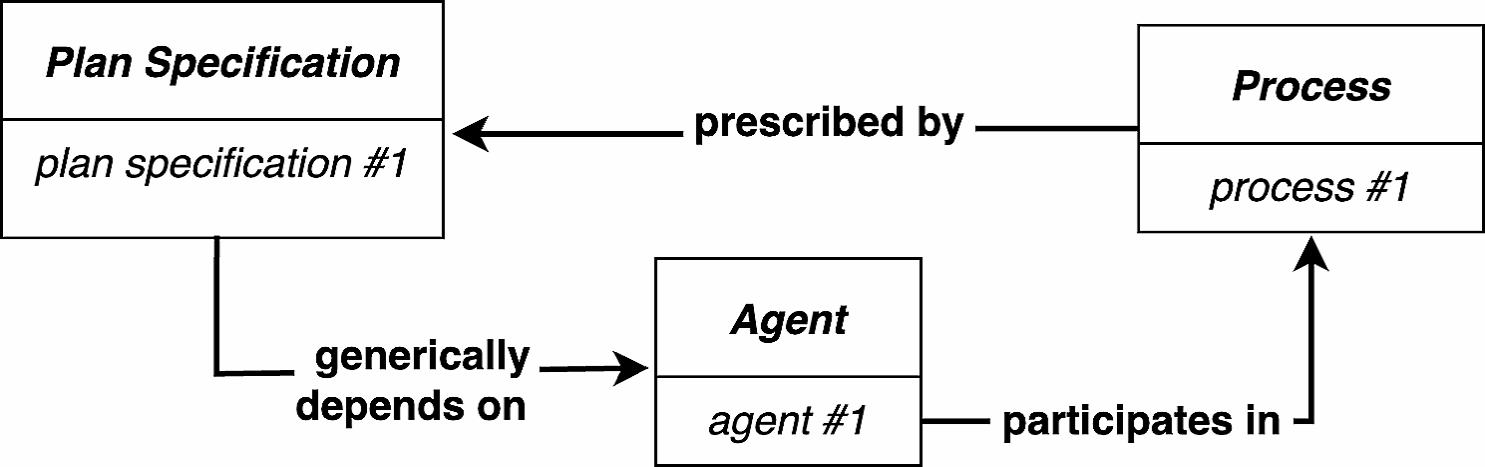
The **prescribed by** relation is used to connect the *Process* to the *Plan Specification* that specifies the actions performed by the *Agent*

CCO, like IAO, is a BFO based ontology, and incorporates IAO’s notion of an *Information Content Entity* (although there are some subtle differences^16^), and this would permit, with suitable harmonization between IAO and CCO, a straightforward way to represent the manner in which a process should unfold (see Fig. 6,^17^).

The RC-account’s difficulty of representing a *Process* that fails to realize a concretized *Plan Specification* is not an issue using the **prescribed by** relation. If the agent fails to carry out the instructions, there is still a clear relation between the *Process* and the *Plan Specification* that was supposed to be executed. In other words, the determination of whether a particular *Process* successfully follows a *Plan Specification* is not tightly bound to the realization of the instance of *Realizable Entity* that **concretizes** the *Plan Specification*. Rather, the level of success is determined by evaluating how well the *Process* conformed to the *Plan Specification*. Moreover, since this approach does not make use of realizable concretizations, we are not faced with the challenge of accounting for the nature of the association between a *Quality* that concretizes a *Plan Specification* and a *Realizable Entity* that concretizes the same *Plan Specification.*

However, there are two disadvantages to this approach. First, some use cases may need to represent changes in an agent’s plans, and, without additional modifications, this is not possible. Second, the **participates in** relation between the agent and the *Process*** prescribed by** the *Plan Specification* is not as direct as it is in the RC-account, and the ability to directly represent which entity executed the *Plan Specification* was one of the important motivations for adopting the RC-account.

### Plan creation process

Our second proposal is to add *Plan* and *Plan Creation Process* classes to IAO:

*Plan* = df A *Disposition* to perform actions aimed at attaining an objective (or objectives) specified within a *Plan Specification*.

*Plan Creation Process* = df A *Process* that **has input**^18^ a concretization of a *Plan Specification* and **results in the formation of**^19^ a *Plan*.

**Fig. 7:**
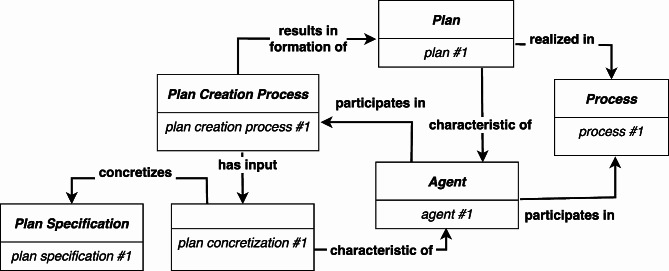
The *Plan Creation* class provides an account of the creation of an agent’s *Plan*

The *Plan* is a **characteristic of** the agent that represents the agent’s intention to carry out the instructions of a *Plan Specification*, and the *Plan Creation Process* uses (or inputs) the concretization of a *Plan Specification* to form the agent’s *Plan* (see Fig. 7,^20^).

In Fig. 7, we have not specified the type that *plan concretization #1* instantiates. This is because in BFO-2020 [8] a *Process* may also concretize a *Generically Dependent Continuant*, and we do not want to restrict *Plan Creation Process* to have only an *SDC* as input.

This approach eliminates the need for the RC-account’s problematic realizable concretization, thus permitting both success and failure to be represented. Since the *Plan* does not directly concretize the *Plan Specification*, a *Plan* may still be realized even though the *Process* that realizes the *Plan* does not attain the objectives of the *Plan Specification*, or the *Plan* is flawed in the sense that it does not accurately reflect the content of the *Plan Specification*. For example, a root canal treatment may fail because the dentist incorrectly performs a step (i.e., a failed execution) or because the dentist forgets to perform a step. In each case, the dentist’s *Plan* (flawed or not) to perform a root canal is a **characteristic of** the dentist and is **realized in** the failed procedure (an instance of *Process*).

**Fig. 8:**
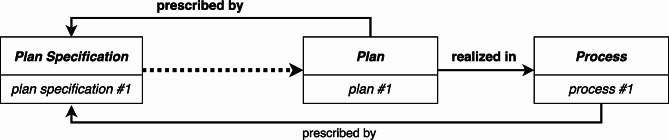
The **prescribed by** relation may be used to specify either the *Plan* that is a **characteristic of** an agent or the process that **realizes** the *Plan*

Although this approach addresses the issues that failures raise for the RC-account, it still lacks an explicit normative relation between a *Plan Specification* and a particular *Plan* (or *Process* that realizes a *Plan*). That is, we have been discussing flawed *Plans* with the assumption that they should implement the actions and goals of the *Plan Specification*. One way to make this normative relation more explicit is incorporate the aforementioned **prescribed by** relation (see Fig. 8).

For example, in the case of the aformentioned root canal procedure, the determination of whether the procedure failed is based on how well it conforms to the *Plan Specification* that **prescribes** how a root canal procedure should be performed. In other words, failure (or success) is not an innate property of a *Plan* or *Process* that realizes a *Plan*. Rather, failure (or success) is a normative judgment of what should happen, not what did happen. In this manuscript, we have not provided an analysis of how such normative judgements occur. We leave this open as a topic for further research.

## Discussion

The aim of this manuscript has been twofold. First, although it is permissible within the IAO framework to represent the concretization of a *Plan Specification* as an instance of *Realizable Entity* (i.e., the RC-account), we wanted to make clear certain issues that arise from the RC-account regarding failures and changes in plans. A defender of the RC-account may counter that it was never intended to represent these situations. The RC-account’s primary purpose was to provide an unambiguous link between the *Plan Specification*, the *Planned Process* during which the instructions were carried out, and the agent that executed the instructions. While we sympathize with that motivation and agree with the RC-account’s goal, the RC-account ignores important realities about failed plans. When medical procedures fail, it is crucial to know if the reason was due to not following the treatment protocol. The process of deliberation often involves deciding to pursue a particular course of action, but later abandoning it to pursue a different course of action. We hold that the inability of the RC-account to adequately represent these real-world phenomena demonstrates that the RC-account should not be used.

Given the shortcomings of the RC-account, the second aim of this manuscript was to provide solutions for representing failures in executing plans and changes in plans. One solution is to make use of the CCO’s **prescribed by** relation. This approach is straightforward and easy to implement. However, the link between the agent’s plan to carry out a set of instructions and the execution of the instructions is not as pronounced as in the RC-account. The other solution introduces the *Plan* and *Plan Creation Process* classes. An instance of *Plan Creation Process* uses the concretization of the *Plan Specification* to form an instance of a *Plan*. The instantiated *Plan* is a **characteristic of** an agent that is realized in a *Process* during which the agent executes the *Plan*. By providing an account for the *Process* that creates a particular *Plan*, the relationship between agent, *Plan* and *Plan Specification* can be represented without raising the issues that stem from the RC-account. We recognize that the second solution is more complex and requires more effort to implement. However, despite its complexity, we hypothesize that the use of the **prescribed by** relation will satisfy many (perhaps most) of the use cases that need to relate the execution of a *Plan Specification* to the process in which it occurs. The more involved *Plan Creation Process* representation proposed by the second solution is reserved for more complex situations that require it. Finally, we point out that our solution also permits the current logical definition of *Planned Process* to be redefined as equivalent to **realizes** some *Plan*, thus retaining the capability of using an automated reasoner to classify a *Process* as being planned.

### Other potential solutions

Our solutions were developed to be consistent with IAO. However, the more recent Core Ontology for Biology and Biomedicine^21^ (COB) [9], Barton et al. [10], and BFO-2020 offer other approaches that may address issues with the RC-account.

#### Core ontology for biology and biomedicine

To account for potential failures when executing a *Plan Specification*, COB redefines *Planned Process* as a *Process* that is intended to realize a *Plan*^22^:

*Planned Process (COB)*: A *Process* that is initiated by an agent who intends to carry out a *Plan* to achieve an objective through one or more actions as described in a *Plan Specification*.

**Fig. 9:**
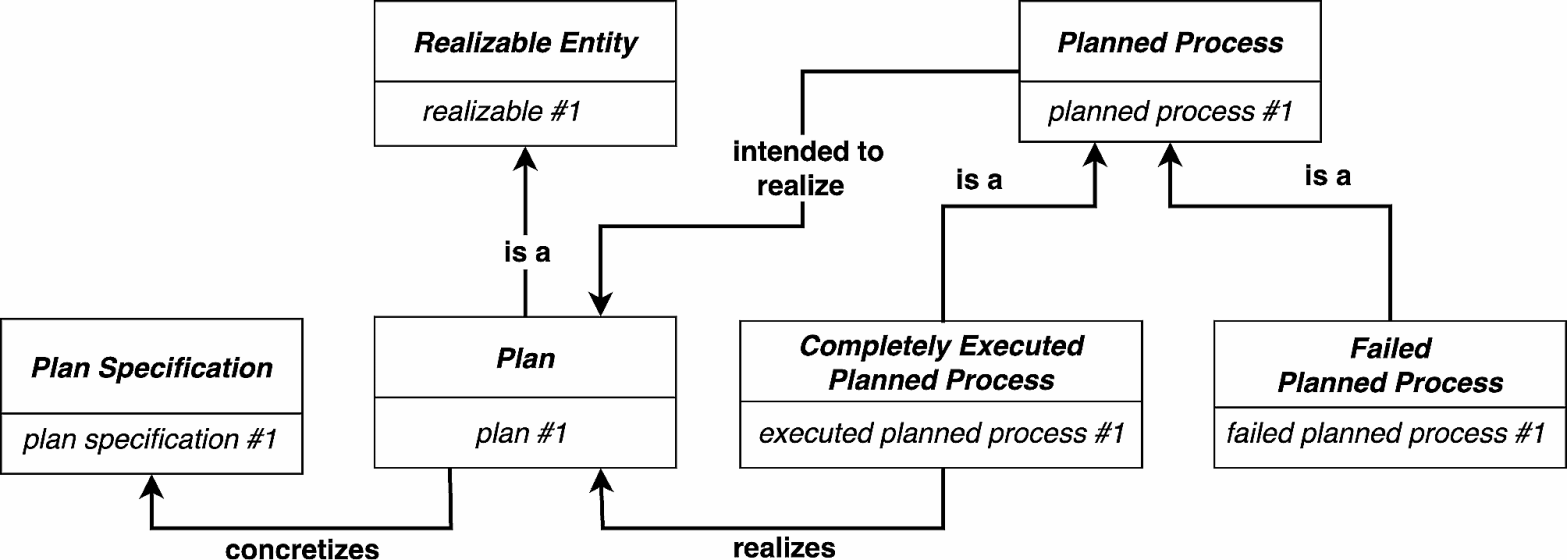
COB distinguishes between *Completely Executed Planned Process* and *Failed Planned Process*

The *Planned Process* hierarchy is then extended to include two subtypes. A *Completely Executed Planned Process*^23^ during which a *Plan*^24^ (a realizable concretization) is realized, and a *Failed Planned Process*, which is not currently defined (see Fig. 9).

The advantage of the COB approach is that, intuitively, it represents the notion that an agent can intend to execute a *Plan*. Sometimes the agent will succeed (resulting in *Completely Executed Planned Process*), and sometimes the agent will fail (resulting in a *Failed Planned Process*). Since only a *Completely Executed Planned Process*** realizes** a *Plan*, it avoids the difficulty in the RC-account for representing a realizable concretization that is** realized in** a failed *Planned Process*. However, it still faces the challenge of representing cases in which the *Plan* is itself flawed, such as in the aforementioned example of when a dentist forgets a step for performing a dental procedure. In such cases, a *Process* may, in fact, realize the flawed *Plan*, and, therefore, it would be classified as a *Completely Executed Planned Process*. Unfortunately, this is counter intuitive, since processes that execute misremembered or incomplete plans are themselves failures (i.e., *Failed Planned Process*).

#### Barton et al. directing actions

In Barton et al., the authors investigate the nature of the relationship between a *Directive Information Content Entity* (*DICE*), which for our purposes is synonymous with IAO’s *Directive Information Entity*, and the action (or actions) that a *DICE* directs an agent to perform.

Similar to COB, they offer a distinction between successfully and attempting to carry out some set of instructions. In their analysis, a particular *DICE d*** s-directs** some *Action a* if *d* “successfully directed” the agent to perform *a*. If the agent was not successful in performing *a*, then *d*** a-directs***a*. Additionally, Barton et al. define a **max-directs** relation account for situations in which an agent is directed to perform a set of actions, such as a recipe that contains multiple steps. For some set of actions *s*, *d*** max-directs ***s* if *d***s-directs** every *Action* that is a member of *s*.

We find Barton et al. lacking on two accounts. First, like COB, there is not an account for those cases in which the agent misunderstands the *DICE*, but successfully carries out the misunderstood action. In such circumstances, did the *DICE*** s-directs** the action, **a-directs** the action, or neither? The answer to this question is not clear. We acknowledge that for simple examples, such as a recipe for baking cookies, this objection may seem far-fetched. However, not all situations are so simple. A *DICE* may communicate complicated actions that require a high-level of understanding – thus, being more subject to misinterpretation – by the agent.

Second, the **s-directs** and **a-directs** relations encompass both the directedness of a *DICE* and conditions of success; that is, whether the actions were successfully completed or attempted. This prevents Barton et al. from providing a clear account of situations in which an agent refuses to perform an action. For example, the agent may find the action to be illegal or immoral. In such cases, the *DICE* may direct a course of action, but it does not make sense to hold that the agent attempted the action. Our account, by contrast, does not have this difficulty. The **prescribed by** relation only serves to provide an account of what should happen and not what did happen.

#### BFO-2020

**Fig. 10:**
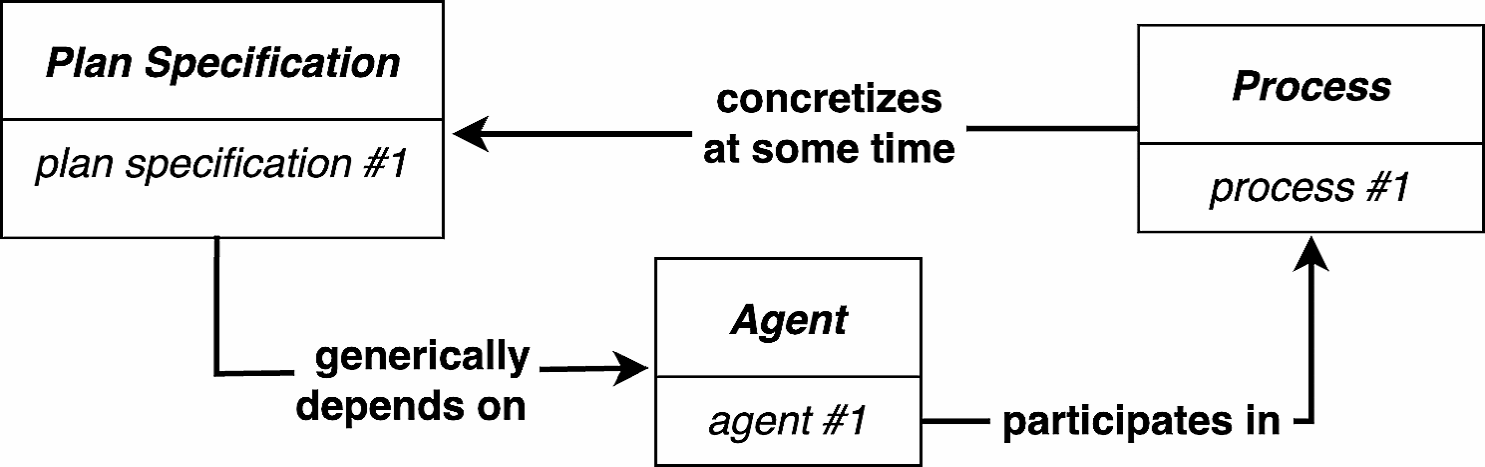
BFO-2020 permits a *Process* to concretize a *Plan Specification*. This may provide a more direct connect between the actions performed during a *Process* and the *Plan Specification* that specify the actions

BFO-2020 now permits a *Generically Dependent Continuant* to be concretized as a *Process*. If IAO were to adopt this updated BFO release, it would open the possibility for a *Process* to concretize *Plan Specification*, thus more directly relating the two entities (see Fig. 10). This approach would be similar to the CCO solution we proposed but would still lack a direct connection between the *Process* and agent offered by the RC-account.

## Conclusions and future research

In this manuscript, we have critiqued the design pattern (or account) for representing the execution of a *Plan Specification* in which a *Process*** realizes** a *Realizable Entity* that **concretizes** the *Plan Specification* (i.e. the RC-account). Our analysis found the RC-account was not adequate for representing situations in which a *Planned Process* fails to bring about the objectives of the *Plan Specification*. When a *Planned Process* fails, it does not, in fact, realize an agent’s intention to execute the *Plan Specification*. This undermines the RC-account’s goal of providing a straightforward way of linking the *Plan Specification*, agent, and *Planned Process* together. Moreover, if the reason for the failure resides in an agent’s misguided intention, we are left with an unsatisfactory account of how an agent’s flawed *Plan*** concretizes** a *Plan Specification*.

Our proposals remedy the RC-account’s shortcomings by decoupling the tight connection between the realization of a *Plan* (i.e., realizable concretization) and the *Process* during which the *Plan Specification* is executed. This permits a *Process* or *Plan* to deviate from the *Plan Specification* without losing their relation to the agent that performs the actions specified by the *Plan Specification*. However, in both of our proposals, it should be noted we have not explicitly represented how an assessment is made as to whether a *Plan* or the *Process* that **realizes** a *Plan* succeeds or fails. Our omission of this is intentional. The execution of a particular *Plan* can both succeed and fail in multiple ways. The agent may have to make subtle alterations to a *Plan* during its execution in order to achieve a specified goal. Thus, the outcome may be deemed a success even though it deviates from the prescribed *Plan Specification*. Similarly, the execution of *Plan* may fail for myriad reasons. For example, environmental factors may prevent an experiment from completing, or ambiguities in the *Plan Specification* may result in the agent performing the wrong actions. Furthermore, there is the issue of representing a *Plan* that partially succeeds (or fails), such as when a *Plan Specification* contains multiple goals and not all the goals are achieved. For these reasons, we decided to forgo the question of how to best represent the assessment of plans (and their executions), and instead focus on issues that arise from the RC-account. The representation of assessing plans is an area for future research.

## Data Availability

No datasets were generated or analysed during the current study.

## Appendix 1: Syntactic conventions and Internationalized Resource Identifier (IRI) table

In order to improve readability, we will adopt the following syntactic conventions when discussing ontology terms. Relations are in bold font (e.g., concretizes, prescribed by), and classes are in italics with the first letter of each word capitalized (e.g., *Plan Specification*, *Planned Process*). Instances of classes (or individuals) are in lowercase italics, and often a hash sign followed by a number is used to uniquely specify individuals (e.g., *plan #1*, *plan #2*). For those ontology terms that have an Internationalized Resource Identifier (IRI), a table mapping the term to its IRI is provided in the table below.

**Table:**

Term	IRI
concretizes	http://purl.obolibrary.org/obo/RO_0000059
prescribes	https://www.ontologyrepository.com/CommonCoreOntologies/prescribes
prescribed by	http://www.ontologyrepository.com/CommonCoreOntologies/prescribed_by
Plan Specification	http://purl.obolibrary.org/obo/IAO_0000104
Study Design	http://purl.obolibrary.org/obo/OBI_0500000
Planned Process (OBI)	http://purl.obolibrary.org/obo/OBI_0000011
Planned Process (COB)	http://purl.obolibrary.org/obo/COB_0000082
Completely Executed Planned Process *	http://purl.obolibrary.org/obo/OBI_0000011
Failed Planned Process	http://purl.obolibrary.org/obo/COB_0000083
Plan (COB)	http://purl.obolibrary.org/obo/OBI_0000260
characteristic of	http://purl.obolibrary.org/obo/RO_0000052
Continuant	http://purl.obolibrary.org/obo/BFO_0000002
Specifically Dependent Continuant	http://purl.obolibrary.org/obo/BFO_0000020
Generically Dependent Continuant	http://purl.obolibrary.org/obo/BFO_0000031
realizes	http://purl.obolibrary.org/obo/BFO_0000055
realized in	http://purl.obolibrary.org/obo/BFO_0000054
intended to realize	http://purl.obolibrary.org/obo/COB_0000081
Process	http://purl.obolibrary.org/obo/BFO_0000015
Quality	http://purl.obolibrary.org/obo/BFO_0000019
Independent Continuant	http://purl.obolibrary.org/obo/BFO_0000004
Occurrent	http://purl.obolibrary.org/obo/BFO_0000003
Realizable Entity	http://purl.obolibrary.org/obo/BFO_0000017
Disposition	http://purl.obolibrary.org/obo/BFO_0000016
Directive Information Entity	http://purl.obolibrary.org/obo/IAO_0000033
has input**	http://purl.obolibrary.org/obo/RO_0002233
results in formation of ***	http://purl.obolibrary.org/obo/RO_0002297

* In the Core Ontology for Biology and Biomedicine (COB), the IRI for *Completely Executed Planned Process* is the same as the IRI for Ontology for Biomedical Investigation’s *Planned Process* class.

** In the Relation Ontology, the range of has input is specified as *Material Entity*. However, in our proposal, the range needs to be expanded.

*** In the Relation Ontology, the range of results in formation of is specified as *Anatomical Entity*. However, in our proposal, the range needs to include *Disposition.*

## Appendix 2: specific and generic dependence

Instances of *Specifically Dependent Continuants* specifically depend on *Independent Continuants*, whereas instances of *Generically Dependent Continuants* generically depend on *Independent Continuants*. If a particular entity *x* specifically depends on another entity (or entities) *y*_*1*_, …, *y*_*n*_, *x* cannot exist unless entities *y*_*1*_, …, *y*_*n*_ exist at every moment that *x* exists. Similar to specific dependence, if *x* generically depends on *y*_*1*_, …, *y*_*n*_, *x* cannot exist unless entities *y*_*1*_, …, *y*_*n*_ exist. However, unlike specific dependence, each *y*_*1*_, …, *y*_*n*_ does not have to exist at every moment *x* exists. For example, suppose *x* generically depends on *y*_*1*_ and *y*_*2*_, and for times *t*_*1*_ and *t*_*2*_, the following hold:

At *t*_*1*_, y_1_ and y_2_ exist.

At *t*_*2*_, y_2_ exists, but *y*_*1*_ does not.

Since *x* generically depends on *y*_*1*_ and *y*_*2*_, *x* exists at both *t*_*1*_ and *t*_*2*_. However, if *x* were to specifically depend on *y*_1_, *x* would have ceased to exist when* y*_1_ ceased to exist.

## Appendix 3: usage of IAO Plan Specification

The following ontologies were extracted from Ontobee.org (https://ontobee.org/ontology/IAO?

*iri =* http://purl.obolibrary.org/obo/IAO_0000104*)* on 2024-05-16. After filtering for OBO Foundry ontologies, 40 ontologies were found to contain IAO’s *Plan Specification* term.

1. Agronomy Ontology.

2. Apollo Structured Vocabulary.

3. Biological Collections Ontology.

4. Chemical Methods Ontology.

5. Coronavirus Infectious Disease Ontology.

6. Cell Line Ontology.

7. Core Ontology for Biology and Biomedicine.

8. CTO: Core Ontology of Clinical Trials.

9. Data Use Ontology.

10. Evidence and Conclusion Ontology.

11. Environment Ontology.

12. Food Ontology.

13. FuTRES Ontology of Vertebrate Traits.

14. Gazetteer.

15. Genomics Cohorts Knowledge Ontology.

16. Genomic Epidemiology Ontology.

17. Health Surveillance Ontology.

18. Hypertension Ontology.

19. Informed consent Ontology.

20. Clinical LABoratory Ontology.

21. Model Card Report Ontology.

22. MIAPA Ontology.

23. Next Generation Biobanking Ontology.

24. Ontology of Biological and Clinical Statistics.

25. Ontology for Biomedical Investigations.

26. Ontology for Biobanking.

27. Oral Health and Disease Ontology.

28. Ontology of Host-Microbiome Interactions.

29. Ontology of Host Pathogen Interactions.

30. Ontology for Modeling and Representation of Social Entities.

31. Ontology for Nutritional Epidemiology.

32. Ontology for Nutritional Studies.

33. Obstetric and Neonatal Ontology.

34. Ontology of Organizational Structures of Trauma centers and Trauma systems.

35. Ontology of Precision Medicine and Investigation.

36. Ontology of RNA Sequencing.

37. The Prescription of Drugs Ontology.

38. Process Chemistry Ontology.

39. Radiation Biology Ontology.

40. Scientific Evidence and Provenance Information Ontology.

41. Space Life Sciences Ontology.

42. The Statistical Methods Ontology.

43. Software ontology.

44. Vaccine Ontology.

## Appendix 4: usage of OBI Planned process

The following ontologies were extracted from Ontobee.org (https://ontobee.org/ontology/OBI?

*iri =* http://purl.obolibrary.org/obo/OBI_0000011*)* on 2024-05-16. After filtering for OBO Foundry ontologies, 61 ontologies were found to contain OBI’s *Planned Process* term.

1. Alzheimer’s Disease Ontology.

2. Agronomy Ontology.

3. Ontology for the Anatomy of the Insect SkeletoMuscular system (AISM).

4. Apollo Structured Vocabulary.

5. Biological Collections Ontology.

6. Chemical Methods Ontology.

7. Coronavirus Infectious Disease Ontology.

8. Cell Line Ontology.

9. Core Ontology for Biology and Biomedicine.

10. CTO: Core Ontology of Clinical Trials.

11. The Drug Ontology.

12. Data Use Ontology.

13. Evidence and Conclusion Ontology.

14. An ontology of core ecological entities.

15. Environmental conditions, treatments and exposures ontology.

16. Environment Ontology.

17. Epilepsy Ontology.

18. Food Ontology.

19. FuTRES Ontology of Vertebrate Traits.

20. Gazetteer.

21. Genomics Cohorts Knowledge Ontology.

22. Genomic Epidemiology Ontology.

23. Genotype Ontology.

24. Health Surveillance Ontology.

25. Hypertension Ontology.

26. Information Artifact Ontology.

27. Informed consent Ontology.

28. Clinical LABoratory Ontology.

29. Medical Action Ontology.

30. Microbial Conditions Ontology.

31. Model Card Report Ontology.

32. Mental Disease Ontology.

33. Next Generation Biobanking Ontology.

34. Ontology of Adverse Events.

35. Ontology of Biological and Clinical Statistics.

36. Ontology for Biobanking.

37. Occupation Ontology.

38. Ontology for General Medical Science.

39. Oral Health and Disease Ontology.

40. Ontology of Host-Microbiome Interactions.

41. Ontology of Host Pathogen Interactions.

42. Ontology for Modeling and Representation of Social Entities.

43. Ontology for Nutritional Epidemiology.

44. Ontology for Nutritional Studies.

45. Obstetric and Neonatal Ontology.

46. Ontology of Organizational Structures of Trauma centers and Trauma systems.

47. Ontology of Precision Medicine and Investigation.

48. Ontology of RNA Sequencing.

49. Ontology of Vaccine Adverse Events.

50. The Prescription of Drugs Ontology.

51. Planaria-ontology.

52. Plant Phenology Ontology.

53. Process Chemistry Ontology.

54. Radiation Biology Ontology.

55. Relation Ontology.

56. Name Reaction Ontology.

57. Scientific Evidence and Provenance Information Ontology.

58. Space Life Sciences Ontology.

59. The Statistical Methods Ontology.

60. Software ontology.

61. Vaccine Ontology.

## Appendix 5: axioms Associated with figures

In order to address ambiguities that may arise from the figures, we include axioms associated with each figure that define how the figure is to be interpreted.

## Appendix 5.1. Axioms for Fig. 2

*plan specification #1*** instance of ***Plan Specification*.

*process #1*** instance of ***Planned Process*.

*agent #1*** instance of ***Agent*.

*concretization #1*** instance of ***Realizable Entity*.

*concretization #1*** concretize**s *plan specification #1*.

*plan specification #1*** generically depends on ***agent #1*.

*concretization #1*** characteristic of ***agent #1*.

*process #1*** realizes ***concretization #1*.

*agent #1*** participates i**n *process #1*.

## Appendix 5.2: axioms for Fig. 4

*restorative treatment specification #1*** instance of ***Plan Specification*.

*root canal specification #1*** instance of ***Plan Specification*.

*restorative concretization #1*** instance of ***Quality*.

*root canal concretization #1*** instance of ***Quality*.

*restorative concretization #1*** concretizes ***restorative treatment specification #1*.

*root canal concretization #1*** concretizes ***root canal specification #1*.

*dentist #1*** instance of ***Dentist*.

*restorative concretization #1*** characteristic of ***dentist #1*.

*root canal concretization #1*** characteristic of ***dentist #1*.

## Appendix 5.3: axioms for Fig. 6

*plan specification #1*** instance of ***Plan Specification*.

*process #1*** instance of ***Process*.

*agent #1*** instance of ***Agent*.

*plan specification #1*** generically depends on ***agent #1*.

*process #1*** prescribed by *** plan specification #1*.

*agent #1*** participates in *** process #1*.

## Appendix 5.4: axioms for Fig. 7

*plan creation process #1*** instance of ***Plan Creation Process*.

*plan specification #1*** instance of ***Plan Specification*.

*process #1*** instance of ***Process*.

*agent #1*** instance of ***Agent*.

*plan #1*** instance of ***Plan*.

*plan concretization #1*** concretizes ***plan specification #1*.

*plan creation process #1*** has input ***plan concretization #1*.

*plan creation process #1*** results in formation of ***plan #1*.

*agent #1*** participates in ***process #1*.

*plan #1*** characteristic of ***agent #1*.

*process #1*** realizes ***plan #1*.

## Footnotes

1 In every case, we refer to the 2022-11-07 version of IAO: http://purl.obolibrary.org/obo/iao/2022-11-07/iao.owl.

2 See **Appendix 1** for a description of the syntactic conventions used in this manuscript and a table of IRIs associated with the ontology terms.

3 Within this manuscript, the term ‘agent’ refers to entities that may carry out a course of action. It is not restricted to humans. Other organisms as well as machines may also be agents.

4 Throughout this manuscript, the non-italics word ‘plan’ is to be understood in the sense of having an intention to carry out a prescribed course of action. We will use the italicized word ‘*plan*’ to refer to either a class (e.g., *Plan*) or instance (e.g., *plan*).

5 https://github.com/CommonCoreOntology/CommonCoreOntologies.

6 An *Information Content Entity* is not restricted to being only about instances.

7 In this example, we use ‘PDF’ in the sense of a computer file that exists as magnetic patterns in memory.

8 https://en.wikipedia.org/wiki/UTF-8.

9 https://en.wikipedia.org/wiki/UTF-EBCDIC.

10 Ontologies in the OBO Foundry are written in the Web Ontology Language (OWL) [6]. The axiom for *Planned Process* is serialized in the Manchester Syntax [7].

11 IAO uses the **characteristic of** relation to represent specific dependence between an *SDC* and an *IC*.

12 For brevity and simplicity, temporal information is not represented; e.g. *concretization #1*** characteristic of*** agent #1* at time *t1.*

13 See **Appendix 5.1** for the axioms that define the interpretation of Fig. 2.

14 For compactness, the **generically depends on** relations between the plan specifications and dentist are not depicted.

15 See **Appendix 5.2** for the axioms that define the interpretation of Fig. 4.

16 In CCO, *Information Content Entity* is generically dependent on an *Information Bearing Entity* and stands in the aboutness relation to some *Entity*. The IRI is also different.

17 See **Appendix 5.3** for the axioms that define the interpretation of Fig. 6.

18 The **has input** relation in this definition does not completely align with the Relation Ontology (RO) term with the same name. The RO term defines the range as *Material Entity*, but in the definition of *Plan Creation Process* the range includes concretizations, which may be instances of *Quality* or even instances of *Process* (see Sect. 4.1.4).

19 The **results in formation of** relation in this definition does not completely align with the Relation Ontology (RO) term with the same name. The RO term defines the range as being *Anatomical Entity*, but in the definition of *Plan Creation Process* the range is *Plan* (a type of *Disposition*).

20 See **Appendix 5.4** for the axioms that define the interpretation of Fig. 7.

21 We reference the 2023-11-26 release of COB (https://purl.obolibrary.org/cob/releases/ 2023-11-16/cob.owl).

22 In COB, *Plan is* taken from OBI (http://purl.obolibrary.org/obo/OBI_0000260) and is defined as a type of *Realizable Entity*. This is different from our *Plan Creation Process Proposal* in which we define *Plan* as a type of *Disposition*.

23 The IRI for COB’s *Completely Executed Planned Process* is the same as OBI’s IRI for *Planned Process* (http://purl.obolibrary.org/obo/OBI_0000011).

24 In this section, *Plan* refers to COB’s term with the IRI http://purl.obolibrary.org/obo/OBI_0000260.

## Abbreviations

BFO: Basic Formal Ontology
CCO: Common Core Ontologies
COB: Core Ontology for Biology and Biomedicine
DICE: Directive Information Content Entity
DIE: Directive Information Entity
IAO: Information Artifact Ontology
IC: Independent Continuant
ICE: Information Content Entity
GDC: Generically Dependent Continuant
OBI: Ontology for Biomedical Investigations
OBO Foundry: Open Biological and Biomedical Foundry
OWL: Web Ontology Language
RO: Relation Ontology
RC: realizable concretization
SDC: Specifically Dependent Continuant
